# Formulation and Characterization of Matrine Oil Dispersion to Improve Droplet Wetting and Deposition

**DOI:** 10.3390/molecules28196896

**Published:** 2023-09-30

**Authors:** Meng Li, Zhen Wang, Huanwen Meng, Dong Wang, Xile Deng, Hongyou Zhou

**Affiliations:** 1 Key Laboratory of Biological Pesticide Creation and Resource Utilization Autonomous Region Colleges and Universities, College of Horticulture and Plant Protection, Inner Mongolia Agricultural University, Hohhot 010020, China; lm1994@emails.imau.edu.cn (M.L.); cau1022@imau.edu.cn (Z.W.); mhw526@126.com (H.M.); wangdong@imau.edu.cn (D.W.); 2 State Key Laboratory of Hybird Rice, Key Laboratory for Biology and Control of Weeds, Hunan Agricultural Biotechnology Research Institute, Hunan Academy of Agricultural Sciences, Changsha 410125, China

**Keywords:** matrine, oil dispersion, formulation, wettability, deposition

## Abstract

The unreasonable use of chemical pesticides has caused serious damage to crops and the ecological environment. The botanical pesticide matrine has attracted attention as an environmentally friendly pesticide. Compared with traditional spraying methods, unmanned aerial vehicle (UAV) spraying has the advantages of safety, rapidity, uniform droplets, low dosages, and no terrain or crop restrictions. In this study, matrine OD was prepared according to the application requirements of flight prevention preparations using three different emulsifiers. The stability, wettability, particle size and distribution, and spraying performance of matrine OD were studied. The results indicated that when the amount of emulsifier was 8%, the three types of matrine OD had good stability. The stability, wettability, particle size and distribution, and spray performance of the suspension prepared using emulsifier VO/03 were better than the other two emulsifiers. Therefore, matrine OD prepared using 8% VO/03 could be used for ultra-low-volume sprays and aerial applications. In this study, we provide a theoretical basis and technical guidance to develop pesticide formulations for aerial applications.

## 1. Introduction

Chemical control remains the primary control measure for agricultural pests and diseases because of its quick effect and high control efficacy [1,2,3,4,5]. The unreasonable use of chemical pesticides has significantly burdened the ecological environment and seriously damaged the ecological balance [6,7,8,9]. The utilization rate of pesticides is only 20–30% because of the splashing, drifting, and evaporation of droplets during spraying; less than 10% reaches the target botanic leaves [10]. The low utilization rate and the enormous loss of pesticides pose a serious threat to the safety of soil and agricultural products. Using traditional spray equipment causes runoff, emissions, dripping, and leaking phenomena, resulting in the loss of pesticide droplets to non-target areas [11,12,13,14,15,16]. Efficient, less toxic, safe, and environmentally friendly botanical pesticides are required to replace chemical pesticides [17,18,19,20,21,22,23,24].

The botanical pesticide matrine is a quinoline pyridine alkaloid. It has good application prospects in medicine and pesticide development [25,26,27,28,29]. In the field of pesticides, matrine is mainly formulated as a soluble concentrate (SL). Although this formulation is safe and convenient to use, its deposition rate of droplets on the surface of plants is poor; thus, it is difficult to achieve an ideal result [30]. Oil dispersion (OD) has recently attracted attention as an efficient and safe new formulation. This formulation has good wettability and spray performance and can significantly improve the deposition of droplets on the target surface to achieve synergistic results [31,32,33]. To obtain the best bioactivity, improve spraying efficiency, and save water and pesticides, it is necessary to use efficient pesticide-spraying technology.

As a new spraying technology, unmanned aerial vehicle (UAV) sprays have the advantages of high efficiency, economy, and safety [34,35,36]. Compared with traditional pesticide-application technologies, UAVs can spray pesticides in fields with different terrains, landforms, and crops of different heights. This significantly reduces the labor intensity of plant protection and improves operation efficiency [37]. A strong penetration force is generated from the high-speed downward rotation and high consistency of the rotor wings to ensure that the liquid rapidly and evenly wets the crops when applying pesticides. This improves the utilization rate of pesticides and saves water and pesticides. The operator is located at a distance from the pesticides and crops; the remote operation of pesticide-application equipment ensures that the pesticide is completely separated, improving the safety of pesticide-application personnel [38,39,40]. Traditional ground spray agents cannot be directly used for UAV spraying, which makes the droplets slip and rebound [41]. 

To develop matrine products suitable for aerial control and improve the application range, application efficiency, and adaptability of matrine, we prepared three types of matrine OD using methyl oleate as the solvent oil and VO/02N, VO/03, and VO/01 as emulsifiers. We evaluated the stability, wettability, and spraying performance of these matrine OD samples. OD samples with good stability and comprehensive performance were screened out; this significantly improved the uniformity of the sprayed pesticides and increased the coverage area of the target surface, thus reducing pesticide use. This may improve the application potential of matrine in aerial spraying.

## 2. Results

### 2.1. OD Formulation and Optimization 

#### Preliminary Screening of the Emulsifier

Emulsifiers are essential additives used to improve the performance and reduce the cost of pesticides. They can ensure not only emulsification but also dispersion and wetting. They help pesticide particles evenly disperse in pesticide systems, prevent the aggregation of pesticide particles, protect suspension systems, and improve the uniformity of pesticides. Emulsifiers can also enhance the stability of pesticide formulations and prolong the residence time of pesticide particles on target crops, thereby strengthening the control effect [42]. An appropriate emulsifier plays a vital role in the performance of OD. In this study, we used VO/02N, VO/03, and VO/01 emulsifier samples. The appearance, pH, and active ingredient content of each sample before and after room temperature (25 °C), cold storage (0 °C ± 1 °C), and hot storage (54 °C ± 2 °C) are listed in Table 1 and Table 2. There were no significant differences between the formulations with emulsifiers VO/02N, VO/03, and VO/01. The decomposition rate of the active ingredients was less than 5%, indicating that the matrine OD samples prepared with these three emulsifiers had high stability. The three emulsifiers were used to prepare the formulation. The follow-up experiments used these three additives as emulsifiers.

### 2.2. Screening of the Thickener Dosage

Thickeners are mainly used as additives to improve the viscosity and rheological properties of pesticide formulations and the persistence and adhesion of pesticide droplets on plant surfaces. They can enhance the performance and stability of pesticides. By strengthening the system’s spatial structure, fluidity, and dispersion, precipitation in the solution is difficult. This improves the effect of pesticides [43]. The optimum amount of thickener directly affects the performance of OD. The changes in our formulation before and after hot and cold storage are listed in Table 3 and Table 4. When the thickener dosages were 3.0%, 2.5%, 2.0%, or 1.5%, the hot-storage stability was qualified, and the active ingredient decomposition rate was less than 5%, the requirements of GB/T 19136-2021 for the thermal storage stability of pesticide preparations were met. The formulation produced a yellow-to-brown turbid liquid and a precipitate that could not be dissolved when the dosage was 1.0%. Therefore, 1.5% was considered the minimum thickener dosage and was used in the subsequent studies.

### 2.3. Effect of Emulsifier Dosage on the Physical Stability of OD

**Figure 1:**
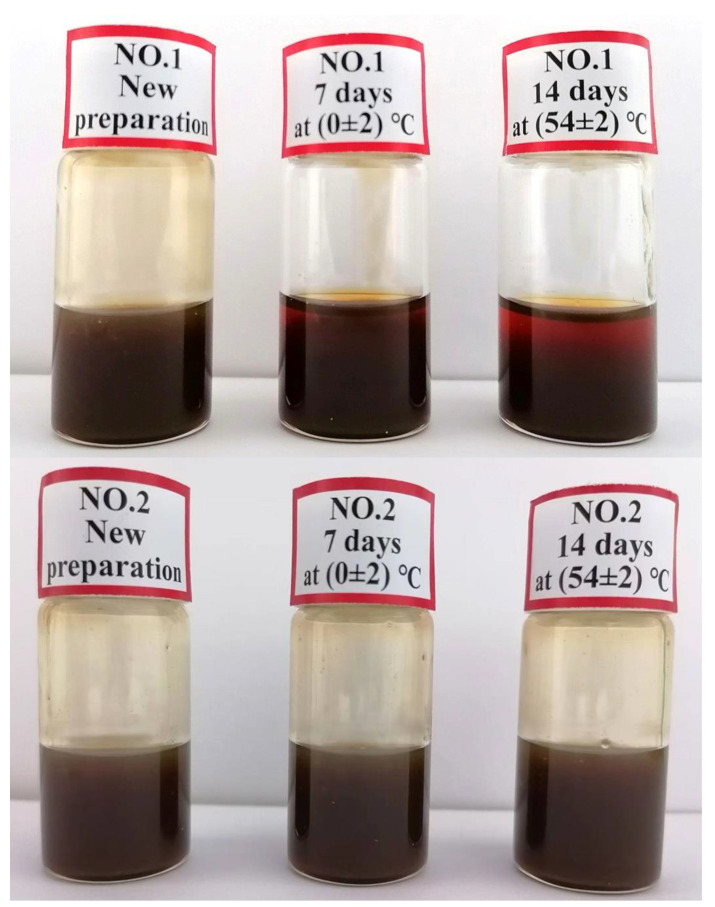
Appearance characteristics of matrine OD samples. As samples 1, 6, and 11 were similar in appearance and the others were similar, only the appearance characteristics of unqualified samples (NO.1) and qualified samples (NO.2) are displayed.

Table 5 and Figure 1 demonstrate that, with the exception of samples 1, 6, and 11 (when the amount of emulsifier was 6%), the appearance of the other 12 samples did not significantly change before and after hot and cold storage. This indicated that the minimum amount required for the three emulsifiers was 8%.

The changes in the pH value and active ingredient content of the 12 samples before and after hot storage are listed in Table 6. After 14 days of storage at 54 ± 2 °C, there were no significant changes in the pH value and active ingredient content of the liquids prepared using different emulsifier dosages; the decomposition rate of active ingredients was less than 5%. A dosage range of 8–15% could be used to prepare matrine OD. The optimum dosage should be determined in combination with other indicators.

### 2.4. Suspensibility

The changes in the suspensibility of each sample before and after 14 days of storage at 54 ± 2 °C are listed in Table 7. The suspensibility of the 12 samples was higher than 95%, which was in line with the relevant provisions of the national standard on the suspensibility of OD [44]. Although the suspensibility of the liquid after hot storage was slightly reduced, it remained higher than 95%. This indicated that the type and dosage of emulsifier had little effect on the suspensibility of matrine OD. Considering the stability and suspensibility results, the amount of these three emulsifiers should be between 8 and 15%. All emulsifiers used met the requirements for the suspensibility of matrine OD.

### 2.5. Multi-Light Scattering Stability

**Figure 2:**
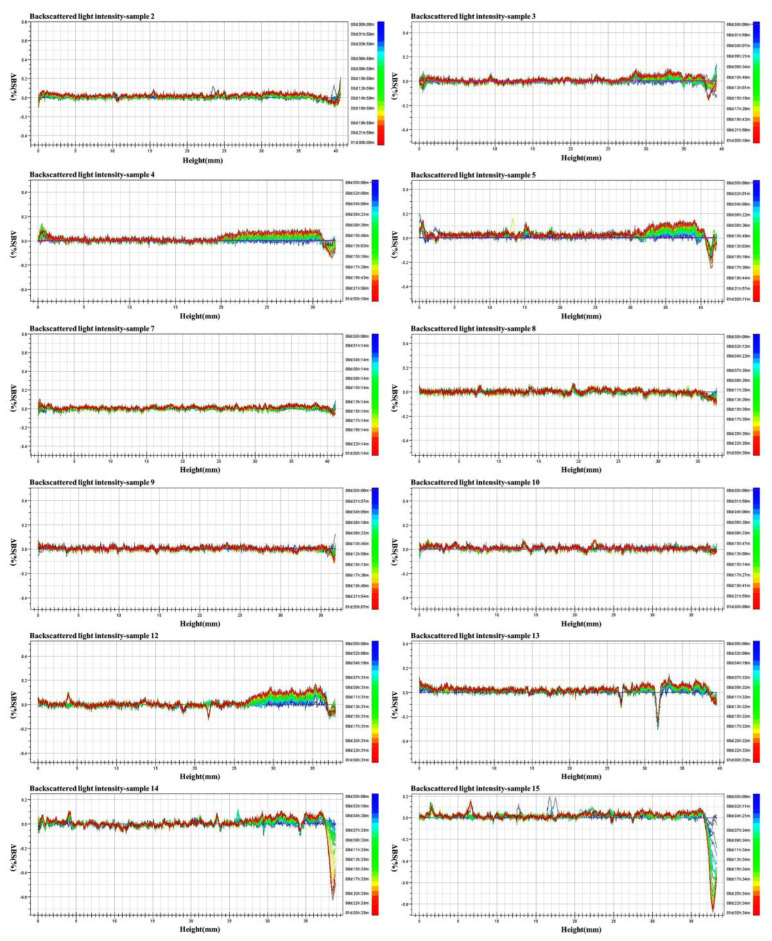
Backscattered light intensity curves of suspensions with different emulsifiers.

Figure 2 demonstrates the real dispersion state of the different experimental samples visually monitored using TurBiscan technology. The backscattered light intensity changed with the time and sample height. The transmittance of all samples was low, which may have been caused by the high viscosity of the suspension. The transmittance of samples 2, 7–10, and 12 was relatively stable in the lower-middle and upper parts (2–35 mm), whereas that of samples 3, 4, and 5 had a slight fluctuation (2–35 mm); that of samples 13, 14, and 15 significantly fluctuated (2–35 mm), but the backscattered light intensity curve changed with time. The results indicated that the prepared OD had good stability, and the performance of OD with four different dosages of VO/03 was the best. 

**Figure 3:**
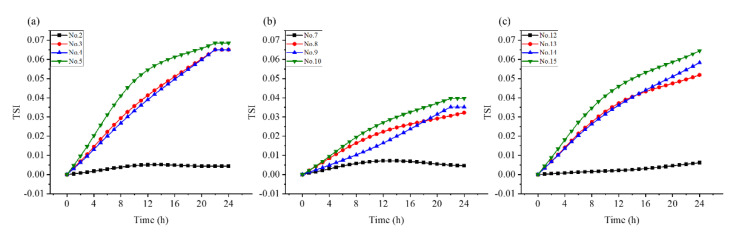
Variations in the TSI values of different emulsifier suspensions with time. ((**a**) No. 2, 3, 4, 5 is the formulation using VO/02N as emulsifier with usage of 8%, 10%, 12%, 15% respectively), ((**b**) No. 7, 8, 9, 10 is the formulation using VO/03 as emulsifier with usage of 8%, 10%, 12%, 15% respectively), ((**c**) No. 12, 13, 14, 15 is the formulation using VO/01 as emulsifier with usage of 8%, 10%, 12%, 15% respectively).

Figure 3 demonstrates that the TSI value of matrine OD prepared with VO/03 was lower than that of OD prepared with the other two emulsifiers. Under the same emulsifier conditions, the smallest TSI value was observed when the emulsifier dosage was 8% (samples 2, 7, and 12). This indicated that the emulsion system had the highest stability at this dosage. The stability of the emulsion decreased with the increase in emulsifier dosage; this could lead to excessive steric hindrance and electrostatic repulsion between particles [45,46].

### 2.6. Determination of the Optimal Formulation 

#### 2.6.1. Static Surface Tension 

**Figure 4:**
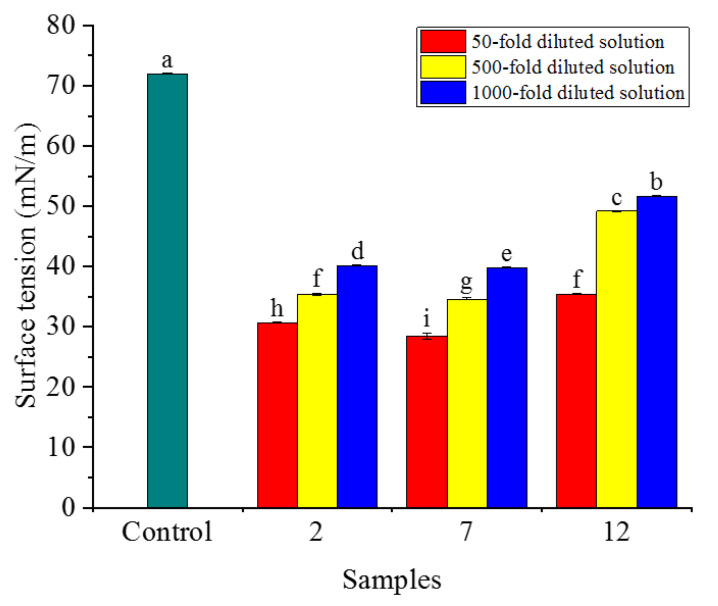
Effect of different doses of the three emulsifiers on SST. Note: Data with different lower-case letters indicate significant differences (*p* < 0.05). Same below.

The wetting of liquid on a solid surface is macroscopically manifested by the attraction of solid surface molecules to liquid molecules. The SST of all samples gradually increased with an increase in the dilution ratio (Figure 4). There were significant differences between samples in the SST of the liquid under the same dilution ratio. Sample 7 (8% VO/03) demonstrated the lowest SST; the wetting area of the sample droplet on the solid surface was the largest, and the hydrophilicity was stronger. When only considering the SST, 8% VO/03 was the most suitable emulsifier for matrine OD.

#### 2.6.2. Dynamic Contact Angle

**Figure 5:**
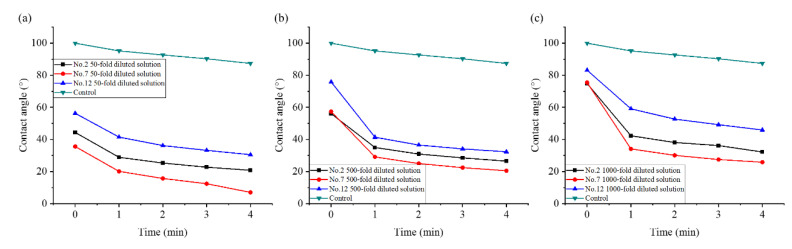
Effect of emulsifier dosage on the DCA: (**a**) 50-fold dilution; (**b**) 500-fold dilution; and (**c**) 1000-fold dilution.

The DCA is an important index used to evaluate the hydrophilicity of a suspension. Figure 5 demonstrates that the DCA of the liquid gradually decreased with the extension of the test time. A decreasing trend of the DCA of each sample was more obvious than that of deionized water. At the same dilution ratio, the initial and equilibrium DCAs (i.e., that at the 4th min) of sample 7 were smaller than those of samples 2 and 12. The wetting hysteresis effect was much smaller than the other two samples, which could wet the solid surface faster. This indicated that the suspension prepared with 8% VO/03 as the emulsifier had better wettability. Considering the effects of the three emulsifiers on the SST, DCA, and viscosity of the liquid, 8% VO/03 was identified as the optimal emulsifier for matrine OD.

#### 2.6.3. Particle Size

**Figure 6:**
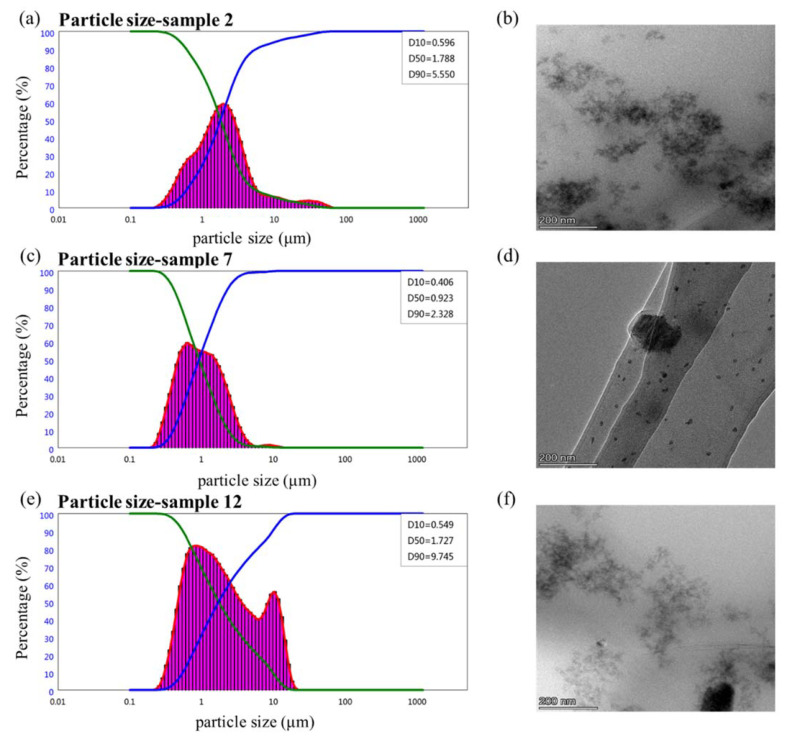
Particle size and distribution of different emulsifier suspensions. (**a**,**b**) is the formulation using 8% VO/02N as emulsifier, (**c**,**d**) is the formulation using 8% VO/03 as emulsifier, (**e**,**f**) is the formulation using 8% VO/01 as emulsifier, the green line is the positive cumulative curve, the blue line is the negative cumulative curve, the purple area is the frequency curve.

Figure 6a,c,e demonstrate that the D_50_ of the three samples was less than 5 μm, indicating that OD met the requirements of the particle size. The D_50_ of sample 7 (0.923 μm) was the smallest; it was much smaller than that of samples 2 (D_50_ = 1.788 μm) and 12 (D_50_ = 1.697 μm). The particle size distribution of sample 7 was similar to a unimodal normal distribution, indicating that the sample had a finer particle size and a more uniform distribution. Figure 6b,d,f demonstrate the TEM results of the suspension particles, indicating that most of the particles in sample 7 were evenly distributed and that the sample had good suspension stability [47]. Based on the multiple light scattering and particle size distribution characteristics of the samples, 8% VO/03 was identified as the most suitable emulsifier for matrine OD.

#### 2.6.4. Spray Performance

**Figure 7:**
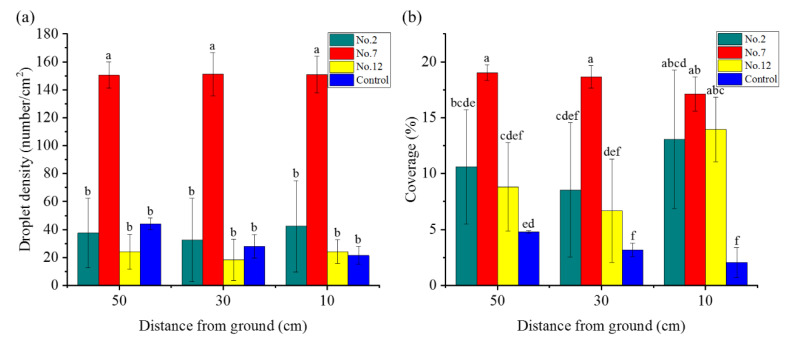
Droplet distribution (**a**) and coverage (**b**) of spray droplets of different emulsifier suspensions.

The droplet depositions of each sample are presented in Figure 7. The average coverage of sample 7 was 18.3%, and the droplet density was 151 numbers/cm^2^. The droplet density and coverage of the suspension on tobacco leaves were significantly higher than those of other treatments and the water controls (except for droplet coverage at 10 cm from the ground). There were no significant differences in the droplet density and coverage rate between the different treatments on tobacco leaves at different heights (except the droplet coverage rate of sample 12 at 10 cm above the ground, which was significantly higher than that at 30 cm), indicating that emulsifier VO/03 could not only improve the droplet density and coverage rate of matrine OD on tobacco leaves but also improve the uniformity of the liquid on leaves at different heights, thereby improving the spray effect of the liquid.

## 3. Conclusions and Discussion

In this study, a specially purposed matrine OD that could be used for aerial pesticide applications was prepared using different emulsifier types and dosages. The optimal emulsifier type and dosage for the formulation were screened using single-factor tests, and the best formulation of matrine OD was obtained. Through the test results of the storage stability, multiple light scattering stability, suspension rate, particle size and distribution, SST, DCA, viscosity, droplet density, and coverage of the formulation, we observed that the trial performance was the best when the dosage of the three emulsifiers was 8%. Emulsifier VO/03 demonstrated the best performance. The optimized formula for matrine OD was determined as follows: 25 g (10%) matrine TC, 20 g (8%) emulsifier (VO/03), 3.75 g (1.5%) organic soil, 5 g (2.5%) white silica, and 250 g solvent oil (methyl oleate). The formulation has broad application prospects, including an ultra-low-volume and drone spray. Compared with the matrine SL developed by Li et al. [48], the use of OD could significantly improve the uniformity of spraying pesticides and increase the coverage area of the target surface, thus providing the possibility of reducing the use of pesticides and reducing the risk of pesticide pollution in the environment. The findings of this study provide a theoretical basis and technical guidance for selecting pesticide formulations and adjuvants, as well as an important strategy for applying oil suspensions in environmentally friendly pesticides. This may also provide a new method for specific formulations suitable for UAV crop protection.

The suspension rate after hot storage was slightly higher than before, which may have been caused by an error during sampling. The specific mechanism and factors require further study.

## 4. Materials and Methods

### 4.1. Materials

We used 80% matrine, purchased from Qingfengyuan Biologics Co., Ltd. (Bayannur, Inner Mongolia, China), and methyl oleate; its main component was *(Z)*-9-octadecenoic acid methyl ester. The solvent was purchased from Zibo Jinghe Biotechnology Co., Ltd. (Shandong, China). The main components of VO/01 were 50% calcium dodecyl benzene sulfate (DBS-Ca) + 50% castor oil polyoxyethylene (BY-110). The main components of VO/02N were 50% calcium dodecyl benzene sulfate + 50% nonylphenol polyoxyethylene ether (OP-7); these two emulsifiers were purchased from Rhodia Feixiang Fine Chemicals Co., Ltd. (Jiangsu, China). The main components of VO/03 were 50% calcium dodecyl benzene sulfate + 50% nonylphenol polyoxyethylene ether (OP-4); this was purchased from Cangzhou Hongyuan Agrochemical Co., Ltd. (Hebei, China). Organic bentonite was used as a thickener; its main component was montmorillonite. Silica was used as a stabilizer; its main component was SiO_2_. The thickener and stabilizer were purchased from Kesai Agrochemical Holdings Co., Ltd. (Shandong, China).

### 4.2. Preparation of Matrine OD 

#### 4.2.1. Preparation Process

According to the following formula ratio (calculated as 250 g), 25 g matrine (8%), 37.5 g emulsifier (15%), 7.5 g organic soil (3%), 5 g white carbon black (2.5%), and solvent oil supplemented to 250 g were evenly sheared and poured into a sand mill (RTSM-0.2BJ, Shanghai Rute Mechanical and Electrical Equipment Co., Ltd., Shanghai, China). Zirconium beads (d = 0.8–1.0 μm) were then added, and the mixture was ground at a speed of 10,000 r/min for 35 min until a particle size of D_50_ ≤ 5 μm was obtained. The mixture was then bottled for later use.

#### 4.2.2. Screening of Emulsifiers

Different emulsifiers were used to prepare matrine OD according to the above formula. After the preparation, the appearance of the liquid was observed at 25 °C for 48 h. The liquids of the different formulations were then maintained at 0 ± 2 °C and 54 ± 2 °C for 7 and 14 days, respectively, to observe the stability and to select the most suitable emulsifier for matrine OD.

#### 4.2.3. Optimization of Thickener Dosage 

According to the formula listed in Section 4.2.1, the thickener dosage was optimized. The specific steps were as follows: matrine OD preparations with organic bentonite dosages of 3, 2.5, 2.0, 1.5, and 1.0% were prepared, and the high (method according to Section 4.3.1) and low (method according to Section 4.3.2) temperature stabilities were tested to screen out the optimum dosage of the thickener.

#### 4.2.4. Emulsifier Dosage Screening 

According to the formula determined in Section 4.2.3, dosage screening and optimization research were further performed. The specific steps were as follows: matrine OD preparations with emulsifier dosages of 15, 12, 10, and 8% were prepared, and the appearance at 25 °C, storage stability (method according to Section 4.3), multiple light scattering stability (method according to Section 4.3.3), static surface tension (SST; method according to Section 4.7.1), and dynamic contact angle (DCA; method according to Section 4.7.2) were tested. The optimal dosage of the emulsifier was determined by considering the stability, SST, and DCA of the formulation.

### 4.3. Stability Test

#### 4.3.1. High-Temperature Stability

The high-temperature stability test was operated according to the China standard GB/T 19136-2021 [49]. A 20 g matrine OD sample was added to the thread sample bottle and placed in an incubator at 54 ± 2 °C for 14 days. The appearance of the matrine OD sample was observed, and the active ingredient content was determined within 24 h. Each sample experiment was repeated four times.

The decomposition rate of the active ingredient was calculated according to Equation (1):

(1) $$c=\frac{a\;-\;b}{a}\;\times \;100\%$$

where a is the effective content before high-temperature storage (%), b is the effective content after high-temperature storage (%), and c is the decomposition rate of the active ingredient (%).

#### 4.3.2. Low-Temperature Stability

The low-temperature stability test was operated according to the China standard GB/T 19137-2003 [50]. A 20 mL matrine OD sample was placed in a threaded sample bottle and stored at 0 ± 1 °C for 7 days. Stratification and precipitation were observed. If there were no such phenomena, it was qualified. Each sample experiment was repeated four times.

#### 4.3.3. Multiple Light Scattering Test 

The multi-light scattering stability of the microemulsion samples was determined using a TurBiscan Lab^Expert^ multi-light scattering instrument (Formulation, Toulouse, France). Approximately 20 mL of an undiluted OD sample was poured into a cylindrical glass tank, and a measuring probe scanned the length of the sample (approximately 40 mm). The intensity of the backscattered light was measured every 40 μm from the bottom to the top of the sample tank. All samples were scanned every hour at 25 °C for 24 h. The stability profile was obtained, and the Turbiscan stability index (TSI) was calculated using TurBiscan Easysoft software. Each sample experiment was repeated three times.

### 4.4. Determination of Suspensibility

According to the China standard GB/T 14825-2006, suspensibility is defined as the amount of active ingredient suspended after a given time in a column (graduated cylinder) of liquid at a stated height expressed as a percentage of the number of active ingredients in the original suspension [49]. The test was repeated three times, and suspensibility was calculated according to Equation (2):

(2) $$\mathrm{Suspensibility}=\frac{10}{9}\times \frac{100(c-Q)}{c}\%=\frac{111(c-Q)}{c}\%$$

where c = ab/100 is the effective content in the prepared suspension sample (g), a is the effective content of the sample determined before or after the sample passed the appropriate accelerated storage (%), b is the mass of the sample (g), and Q is the mass of effective content in the 10 mL suspension left in the graduated cylinder (g).

### 4.5. Determination of Viscosity 

An NDJ/SNB series rotary viscometer (Shanghai Jinghai Instrument Co., Ltd., Shanghai, China) (28# rotor) was used to measure the viscosity. The prepared matrine OD sample was poured into the sample cell, and the speed was adjusted to 40 r/min. When the reading was stable, the viscosity was measured three times in parallel, and the average value was obtained [51].

### 4.6. Particle Size Determination 

The particle size was measured using a BT-9300S laser particle size analyzer (Liaoning Dandong Baite Instrument Co., Ltd., Liaoning, China). The process was repeated five times, and the D_50_ value was recorded. 

The microscopic characteristics of the suspension and the distribution of the pesticide particles in the pesticide solution were analyzed using a transmission electron microscope (TEM; TALOS F200X, Shanghai Freescale Technology Co., Ltd., Shanghai, China).

### 4.7. Determination of Wettability 

#### 4.7.1. Static Surface Tension 

Matrine OD was diluted with deionized water into 50-fold, 500-fold, and 1000-fold aqueous diluents. The SST of the diluents was measured using an optical contact angle measuring instrument (DSA-100, KRUSS, Hamburg, Germany). Each sample was measured three times and averaged. The test temperature was 20 ± 1 °C.

#### 4.7.2. Dynamic Contact Angle 

For a high utilization rate, pesticides must be rapidly spread and deposited on the leaf surface of the target plant. This requires good wettability of the liquid droplets. The common method to determine the wettability of pesticide droplets is to determine the DCA of droplets on a solid surface [52,53]. Matrine OD was diluted with deionized water into 50-fold, 500-fold, and 1000-fold aqueous diluents. A drop of diluent was taken using a needle with an inner diameter of φ = 0.8 mm, and a drop was dropped every 5 μL. The DCA was measured every second using an optical contact angle measuring instrument (DSA-100, KRUSS, Germany). The DCA was measured for 0–4 min, repeated four times at 20 ± 1 °C, and photographs of the droplet shapes were taken at the 0, 1st, 2nd, 3rd, and 4th min.

### 4.8. Determination of Droplet Density 

Droplet density was measured using water-sensitive test paper. Three 3 × 4 cm water-sensitive test papers were fixed on tobacco leaves at different heights (10, 30, and 50 cm from the ground), and the matrine diluent was sprayed with an UAV (TH40, Shandong Tonghui Intelligent Technology Co., Ltd., Shandong, China) at a volume of 50 mL/667 m^2^. The spray height was 1.5 m. After the application, the test paper was scanned using a scanner (DJI MG-1/S/A/P, Shenzhen DJI Innovation Technology Co., Ltd., Shenzhen, China) [54], and the droplet deposition characteristics and coverage were calculated using scanner-equipped droplet analysis software (V1.2.4).

## Figures and Tables

**Table 1:** Effect of emulsifier type on the stability of the formulation.

Emulsifier	Use Level	Storage Time (Temperature)				
		0 h (25 °C)	24 h (25 °C)	48 h (25 °C)	7 d (0 °C ± 1 °C)	14 d (54 °C ± 2 °C)
VO/02N	15%	Homogeneous	Homogeneous	Homogeneous	Homogeneous	Homogeneous
VO/03	15%	Homogeneous	Homogeneous	Homogeneous	Homogeneous	Homogeneous
VO/01	15%	Homogeneous	Homogeneous	Homogeneous	Homogeneous	Homogeneous

**Table 2:** Effect of emulsifier type on the formulation response to hot storage.

Emulsifier	Use Level	pH		Active Ingredient Content (%)		
		Before Hot Storage	After Hot Storage	Before Hot Storage	After Hot Storage	Rate of Dissociation (%)
VO/02N	15%	6.69	6.76	8.24	8.10	1.70
VO/03	15%	6.48	6.50	8.89	8.67	2.47
VO/01	15%	7.84	7.79	8.62	8.28	3.94

**Table 3:** Effect of organic soil thickener dosage on the stability of the formulation.

Emulsifier Levels	Dosage of Thickener	Appearance before Cold Storage	Appearance after Cold Storage
15% VO/02N 15% VO/03 15% VO/01	3.0%	Homogeneous	Homogeneous
	2.5%		
	2.0%		
	1.5%		
	1.0%	Produced turbid liquid with precipitate; the precipitation did not disappear after oscillation	

**Table 4:** Effect of thickening agent content on the high-temperature stability of the formulation.

Mulsifier Levels	Use of Thickening Agent	pH		Active Ingredient Content (%)			Appearance	
		Before Hot Storage	After Hot Storage	Before Hot Storage	After Hot Storage	Rate of Dissociation	Before Hot Storage	After Hot Storage
15% VO/02N 15% VO/03 15% VO/01	3.0%	6.69	6.76	8.14	8.10	0.005	Homogeneous	Homogeneous
	2.5%	7.34	6.69	8.20	8.09	0.01		
	2.0%	6.54	6.13	8.16	8.06	0.01		
	1.5%	6.03	5.90	8.24	8.17	0.008		
	1.0%	5.89	5.82	8.14	8.06	0.01	Turbid	

**Table 5:** Effect of emulsifier dosage on the stability of the formulation.

NO.	Emulsifier	Storage Time (Temperature)				
		0 h (25 °C)	24 h (25 °C)	48 h (25 °C)	7d (0 °C ± 1 °C)	14 d (54 °C ± 2 °C)
1	6% VO/02N	Turbid	Turbid	Turbid	-	-
2	8% VO/02N	Grey liquid	Homogeneous	Homogeneous	Homogeneous	Homogeneous
3	10% VO/02N	Grey liquid	Homogeneous	Homogeneous	Homogeneous	Homogeneous
4	12% VO/02N	Grey liquid	Homogeneous	Homogeneous	Homogeneous	Homogeneous
5	15% VO/02N	Grey liquid	Homogeneous	Homogeneous	Homogeneous	Homogeneous
6	6% VO/03	Turbid	Turbid	Turbid	-	-
7	8% VO/03	Grey liquid	Homogeneous	Homogeneous	Homogeneous	Homogeneous
8	10% VO/03	Grey liquid	Homogeneous	Homogeneous	Homogeneous	Homogeneous
9	12% VO/03	Grey liquid	Homogeneous	Homogeneous	Homogeneous	Homogeneous
10	15% VO/03	Grey liquid	Homogeneous	Homogeneous	Homogeneous	Homogeneous
11	6% VO/01	Turbid	Turbid	Turbid	-	-
12	8% VO/01	Grey liquid	Homogeneous	Homogeneous	Homogeneous	Homogeneous
13	10% VO/01	Grey liquid	Homogeneous	Homogeneous	Homogeneous	Homogeneous
14	12% VO/01	Grey liquid	Homogeneous	Homogeneous	Homogeneous	Homogeneous
15	15% VO/01	Grey liquid	Homogeneous	Homogeneous	Homogeneous	Homogeneous

**Table 6:** Effect of emulsifier dosage on the hot-storage stability of the formulation.

NO.	pH		Active Ingredient Content (%)		
	Before Hot Storage	After Hot Storage	Before Hot Storage	After Hot Storage	Rate of Dissociation (%)
2	7.50	7.36	8.25	8.09	1.94
3	6.94	7.04	8.21	8.09	1.46
4	6.72	6.80	8.26	8.17	1.09
5	6.69	6.76	8.24	8.10	1.70
7	6.76	6.76	8.18	8.09	1.10
8	6.68	6.65	8.16	8.02	1.72
9	6.55	6.59	8.12	8.04	0.99
10	6.48	6.50	7.89	7.67	2.79
12	7.74	7.75	8.98	8.74	3.44
13	7.32	7.41	8.12	8.03	1.26
14	7.45	7.46	8.80	8.65	1.92
15	7.84	7.79	8.62	8.58	0.52

**Table 7:** Effect of emulsifier dosage on the suspension rate of the formulation.

No.	Suspensibility (%)	
	Before Hot Storage	After Hot Storage
2	99.09	98.25
3	99.67	99.03
4	97.69	97.19
5	98.02	96.71
7	99.33	98.89
8	98.97	98.64
9	96.32	96.29
10	99.64	97.81
12	99.17	98.64
13	99.08	98.76
14	99.69	98.73
15	98.55	97.85

## Data Availability

Data are available within the article.

## Sample Availability

Samples of the compounds are available from the authors.

## Abbreviations

TC: technical material; OD: oil dispersion; SL: soluble concentrate; DCA: dynamic contact angle; SST: static surface tension; TSI: Turbiscan stability index; UAV: unmanned aerial vehicle.
